# Widespread occurrence of chromosomal aneuploidy following the routine production of *Candida albicans* mutants

**DOI:** 10.1111/j.1567-1364.2009.00563.x

**Published:** 2009-09-01

**Authors:** Mélanie Arbour, Elias Epp, Hervé Hogues, Adnane Sellam, Celine Lacroix, Jason Rauceo, Aaron Mitchell, Malcolm Whiteway, André Nantel

**Affiliations:** 1 Biotechnology Research Institute, National Research Council of CanadaMontreal, QC, Canada; 2 Department of Biology, McGill UniversityMontreal, QC, Canada; 3 Department of Anatomy and Cell Biology, McGill UniversityMontreal, QC, Canada; 4 Department of Sciences, John Jay College of Criminal JusticeNew York, NY, USA; 5 Department of Biological Sciences, Carnegie Mellon UniversityPittsburgh, PA, USA

**Keywords:** *Candida albicans*, aneuploidy, karyotype, chromosome structure, microarray

## Abstract

It has come to our attention that approximately 35% of >100 published microarray datasets, where transcript levels were compared between two different strains, exhibit some form of chromosome-specific bias. While some of these arose from the use of strains whose aneuploidies were not known at the time, in a worrisome number of cases the recombinant strains have acquired additional aneuploidies that were not initially present in the parental strain. The aneuploidies often affected a different chromosome than the one harboring the insertion site. The affected strains originated from either CAI-4, RM1000, BWP17 or SN95 and were produced through a variety of strategies. These observations suggest that aneuploidies frequently occur during the production of recombinant strains and have an effect on global transcript profiles outside of the afflicted chromosome(s), thus raising the possibility of unintended phenotypic consequences. Thus, we propose that all *Candida albicans* mutants and strains should be tested for aneuploidy before being used in further studies. To this end, we describe a new rapid testing method, based on a multiplex quantitative PCR assay, that produces eight bands of distinct sizes from either the left or right arms of each *C. albicans* chromosome.

## Introduction

We wish to warn the *Candida albicans* research community that cases of aneuploidy in *C. albicans* laboratory strains, first identified by Chen *et al.* (2004) and reviewed extensively by Rustchenko (2007), are commonly exacerbated following routine genetic manipulations such as gene insertions and inactivations.

*Candida albicans* is an opportunistic fungal pathogen with a diploid genome and an incomplete sexual cycle. While its genome sequence was first released in 2000, published in 2004 (Jones *et al.*, 2004) and annotated in 2005 (Braun *et al.*, 2005), the lack of detailed physical and genetic maps have delayed the production of a final chromosomal assembly by several years (Nantel, 2006). Nevertheless, researchers using techniques such as pulse-field gel electrophoresis and contour-clamped homogeneous electrical field (CHEF) gels were already amassing a significant body of evidence demonstrating chromosomal instability in *C. albicans* under laboratory conditions (Rustchenko, 2007). For example, cells whose sole source of carbon was l-sorbose tended to lose a copy of chromosome 5 (Chr 5) while growth in d-arabinose promoted Chr 6 trisomy (Rustchenko *et al.*, 1994; Kabir *et al.*, 2005;). Chen *et al.* (2004) observed the loss of one copy of Chr 1 in strains exposed to 5-fluoroorotic acid. They also demonstrated that the commonly used laboratory strains CAI-4 and SGY-243 carried an extra copy of Chr 1 and that this triploidy had a negative effect on virulence. Comparative genome hybridization (CGH) studies conducted by Selmecki *et al.* (2005) identified an additional, albeit unstable, Chr 2 aneuploidy in CAI4 as well as a heterozygous deletion in the right arm of Chr 5 in RM1000 and its derivative BWP17. The recent release of the *C. albicans* genome assembly by van het Hoog *et al.* (2007) has permitted more detailed studies including the identification of a Chr 5 isochromosome linked to azole resistance (Coste *et al.*, 2006; Selmecki *et al.*, 2006;). Finally, it was suggested by Ahmad *et al.* (2008) that different stock of the same *C. albicans* laboratory strain may harbor different types of chromosomal aberrations. All of these results suggest that the *C. albicans* genome has enough plasticity to support a wide variety of different chromosomal aneuploidies and that changes in chromosome copy numbers often arise as a response to stress.

## Materials and methods

### Analysis of microarray data

*Candida albicans* transcriptional profiling data were extracted from a variety of sources and visualized using the genespring gx v7.3 (Agilent Technologies) physical view. Alternatively, fluorescence data on target genes were sorted in Microsoft Excel according to their chromosomal map coordinates and visualized on a scatter plot.

### CGHs

*Candida albicans* genomic DNA was isolated from a saturated overnight culture with the Qiagen Genomic DNA Extraction kit and labeled with either Cy3 or Cy5 dyes with the Bioprime CGH Labeling (Invitrogen). Unincorporated nucleotides were removed with Qiagen PCR columns and the labeled probes were then hybridized as described to DNA microarrays spotted with 6354 70mer oligonucleotide probes representing most of the genes identified in Genome Assembly 19 (Nantel *et al.*, 2006). Normalization and data analysis were performed in genespring gx v7.3 (Agilent Technologies). When used to validate suspected aneuploidies, most GCH experiments needed to be performed only once. Because our microarray probes are randomly distributed, it is impossible for noise, dye bias or probe localization artifacts to produce a fluorescence ratio bias that is specific only to certain chromosomes. Thus, even a very weak change in median fluorescence ratio becomes very obvious when viewed in this chromosomal context.

### Multiplex PCR

The genomic DNA was purified by the Yeast Smash & Grab DNA miniprep method as described by Rose *et al.* (1990). For multiplex PCR, we used the Qiagen multiplex PCR kit. PCR reaction mixtures (total volume, 50 μL) contained 1 × Qiagen multiplex PCR master mix, 0.125 μM equimolar primers mixture (either A or B, see Table 1) and 1–50 ng of purified genomic DNA (the specific amount must be evaluated by each individual lab). Thermal cycling was carried out in a Thermocycler 9600 (Perkin-Elmer) with a denaturation step of 95 °C for 15 min, 23 cycles with 30 s denaturation at 94 °C, 30 s annealing at 57 °C, 45 s elongation at 72 °C and the last elongation step at 72 °C for 7 min. When performed to validate an already known aneuploidy, the PCR assays needed to be performed only once to validate the results. When used for screening, replicate experiments on several individual colonies are advisable.

**Table 1:** Primer sequences used in the multiplex PCR aneuploidy detection assay

	Primer set A (left arm)			Primer set B (right arm)		
Chr	Sequences	Positions	Amplicon lengths	Sequences	Positions	Amplicon lengths
1	Ca21Chr1_A_L acttgtacggctggaaaaact	21272	301	Ca21Chr1_B_L caactgccaaactagttccaa	3155855	305
	Ca21Chr1_A_R gccaagtatgagagggttgat	21572		Ca21Chr1_B_R tgttggtgttttaccgtgttt	3156159	
2	Ca21Chr2_A_L cgagttaaactttcggtttcc	15481	383	Ca21Chr2_B_L tccttctggcccttctaagta	2213805	375
	Ca21Chr2_A_R attgagggattgaacaaggag	15863		Ca21Chr2_B_R aagagtgagcttgttctgggt	2214179	
3	Ca21Chr3_A_L atgctcctgtaatacgctcct	38238	478	Ca21Chr3_B_L catgttttagttggtcgatgg	1779058	471
	Ca21Chr3_A_R gctcacacaatccaaccatag	38715		Ca21Chr3_B_R gtaaccgacaaactccatgtg	1779528	
4	Ca21Chr4_A_L cacagagatgacagaacaccc	6565	588	Ca21Chr4_B_L gatttgcggtggtttattttt	1614471	593
	Ca21Chr4_A_R cttgatccccaccatagactt	7152		Ca21Chr4_B_R aaactagtctaccctgccgaa	1615063	
5	Ca21Chr5_A_L tgacaacattggagatggtct	28726	472	Ca21Chr5_B_L cggtcatgtatttgattacgg	1163697	741
	Ca21Chr5_A_R agatttcgaatcacgcttttt	29467		Ca21Chr5_B_R tatctgcagacgactacccag	1164437	
6	Ca21Chr6_A_L acatcatcctgtaacgccata	13849	925	Ca21Chr6_B_L tgcgtctagatacaacaaggc	1014943	917
	Ca21Chr6_A_R caggtcaactcaacttccaga	14773		Ca21Chr6_B_R acttggcatcaacttccttct	1015859	
7	Ca21Chr7_A_L gtcattccgaatctcaaacct	4219	1153	Ca21Chr7_B_L aagtatgcaatttctttgggg	931287	1151
	Ca21Chr7_A_R tgaaaagtgcaggagaatcac	5371		Ca21Chr7_B_R tcctcagcctgtttgtagttg	932437	
R	Ca21ChrR_A_L ccaatataccccaatccaaac	18490	1430	Ca21ChrR_B_L atttggtagaagatcgatggg	2287349	1438
	Ca21ChrR_A_R aaagacttgttccacctcacc	19919		Ca21ChrR_B_R aagacaacaacgaagatgctg	2288786	

### Agilent 2100 Bioanalyzer microcapillary electrophoresis

Following amplification, 1 μL of the PCR reaction was loaded into the well of a Bioanalyzer chip prepared according to manufacturer's protocol for the DNA 7500 Lab Chips (Agilent Technologies). The aneuploidy of the mutant DNA was determined by the relative ratio of the peak height of the mutant and wild-type (SC5314) DNA fragments in the chromatogram. The ratio for each chromosome was then divided by the median of the ratio for all chromosomes. Alternatively, the elution profile graphics were scaled and overlapped in an image processing software such as abode photoshop.

## Results

**Fig. 1:**
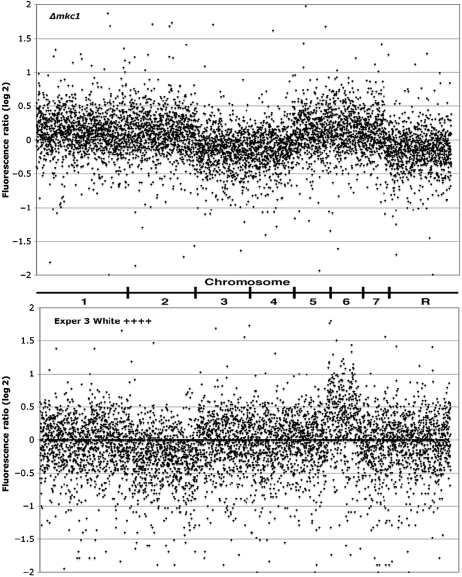
Example of chromosomal bias in published transcriptional profiles. Each spot represents fluorescence ratio data (log 2) from genes that were ordered according to their position on the eight *Candida albicans* chromosomes. The top panel represents a comparison between a Δ*mkc1* strain and its *CAI4* parental strain (Oberholzer *et al.*, 2006). The bottom panel shows the profile obtained from a comparison between a ‘white’ morphology strain expressing the α1, α2, a1 and a2 *MTL* transcriptional regulators and its *CAI4* parental strain at 23°C (Tsong *et al.*, 2003).

Following the completion of the *C. albicans* genome assembly (van het Hoog *et al.*, 2007), we noticed that some of our transcriptional profiles exhibited chromosome-specific bias. Thus, we extended this analysis to >100 published and unpublished microarray experiments where two different strains, usually a mutant and its wild-type parent, were directly compared. As reported in Table 2, we observed cases of chromosomal bias in 22 out of 59 *C. albicans* strains (36.2%). For example, in Fig. 1, we can clearly see that the transcriptional profiles obtained by comparing a *Δmkc1* recombinant strain (Oberholzer *et al.*, 2006) to the SC5314 control strain show an enrichment in overexpressed genes that are located in Chr 1, 2, 5, 6 and 7 while the genes in Chr 3, 4 and R tend to be repressed. While the actual fold-change can be minor, the bias can be easily detected, as long as the probes are spotted in a random position on the microarray slides so that experimental noise and most changes in transcript abundance are evenly distributed. The second example, taken from Tsong *et al.* (2003), is especially interesting because we can see both up- and downregulated chromosome bias; the general repression of Chr 2 genes is due to the use of the Chr 2 trisomic CAI4 strain as a control, while the increase in the fluorescence ratios of genes in Chr 6 is the probable result of a chromosome duplication event during the generation of this strain.

**Table 2:** Identification of unexpected aneuploidies in microarray profiles

Mutation	Function	Gene locations	Aneuploidy	Mode of production (reference if different from array data)	Reference for array data
D9-330	Antifungal resistance	N/A	Isochromosome 5* directed evolution	Cowen *et al.* (2002)	
D11-330	Antifungal resistance	N/A	Gain of Chr 7*	Directed evolution	Cowen *et al.* (2002)
D12-165	Antifungal resistance	N/A	Gain of Chr 7*	Directed evolution	Cowen *et al.* (2002)
D12-330	Antifungal resistance	N/A	Gain of Chr 4*	Directed evolution	Cowen *et al.* (2002)
Δ*efg1/cph1*	Transcription factors	Chr R and Chr 1	Gain of Chr 7‡	Ura-blaster (Lo *et al.*, 1997)	Nantel *et al.* (2002)
35/65 profiles	Combinations of MTL transcriptional regulators in White and Opaque cells	Various	Mixed cell population, some with gain of either Chr 6 or 7. Use of CAI4 as control also caused Chr 2 bias in 33 of the comparisons	PCR disruption cassettes	Tsong *et al.* (2003)
Δ*cdc35*	Adenylate Cyclase	Chr 7	Gain of Chr 2‡	Ura-blaster (Rocha *et al.*, 2001)	Harcus *et al.* (2004)
Δ*ssn6*	Transcription factor	Chr 3	Gain of Chr 7	Ura-blaster	Garcia-Sanchez *et al.* (2005)
Δ*mkc1*	MAP kinase	Chr R	Loss of Chr 3, 4 and R	PCR disruption Cassette	Oberholzer *et al.* (2006)
Δ*sst2*	GTPase activator	Chr 5	Gain of Chr 6	PCR disruption Cassette	Dignard & Whiteway (2006)
Δ*tac1*	Transcription factor	Chr 5	Loss of Chr 5†	MPA^R^-flipping	Znaidi *et al.* (2007)
*TAC1* replacements	Transcription factor	Chr 5	Gain of Chr 5 and/or 7†	MPA^R^-flipping	Znaidi *et al.* (2007)
Δ*cph1*	Transcription factor	Chr 1	Gain of Chr 6 and 7‡	Ura-blaster (Lo *et al.*, 1997)	Huang *et al.* (2008)
Δ*ras1*	Small GTPase	Chr 2	Gain of Chr 4 and loss of Chr 5–6	hph-URA3-hph disruption cassette (Feng et al., 1999)	B. Hube, GSE11490
Δ*ste4*	G® subunit	Chr 2	Loss of Chr 1-3	Ura-blaster	
Δ*rds2*	Transcription factor	Chr R	Loss of Chr 1	Ura-blaster	B. Turcotte (unpublished data)
Δ*fun31*	Ser/Thr protein kinase	Chr 3	Loss of Chr R	PCR disruption Cassette	J. Rauceo and A. Mitchell (unpublished data)
Δ*vma22*	Membrane protein	Chr R	Loss of right arm of Chr R†	PCR disruption Cassette	E. Epp and M. Whiteway (unpublished data)
Δ*nrg1*	Transcription factor	Chr 7	Gain of Chr 2 and 4†‡	Ura-blaster (Murad et al., 2001)	C. Lacroix and A. Nantel (unpublished data)
HA-Rfg1	Tagged transcription factor	Chr 1	Gain of Chr 1 and/or Chr 2†‡	Insertion in *Rps1* site	C. Lacroix and A. Nantel (unpublished data)
Nrg1-HA	Tagged transcription factor	Chr 1	Gain of Chr 2†‡	Insertion in *Rps1* site	C. Lacroix and A. Nantel (unpublished data)

Note that the ‘loss’ of a chromosome might also be indicative of duplication of the remaining chromosomes or the presence of a trisomic chromosome in the control strain.

Following a review of available data, profiles for the following mutants did not seem to exhibit obvious aneuploidies: Δace2, Δ*ada2*, Δ*als2*, Δ*als4*, Δ*bcr1*, Δ*bub2*, Δ*cdc5*, Δ*cdc53*, Δ*cdr1*Δ*cdr2*, Δ*cek1*, Δ*cka2*, Δ*crz1*, Δ*cph1*Δ*efg1*, Δ*cst20*, Δ*dfg16*, Δ*efg1*, Δ*efg1*Δ*efh1*, Δ*efh1*, Δ*gal4*, Δ*gcn2*, Δ*gcn4*, Δ*hog1*, Δ*hst7*, Δ*msn4*Δ*mnl1*, Δ*myo5*, Δ*myo5*Δ*hog1*, Δ*myo5*Δ*mkc1*, Δ*myo5*Δ*SH3*Δ*A*, Δ*rfg1*, Δ*rim101*, Δ*rlm1*, Δ*sit4*, Δ*sla2*, Δ*ssr1*, Δ*tup1*, D8-330, N4-330.

* Observation validated by CGH (Selmecki *et al.*, 2006; A. Selmecki, L.E. Cowen and J. Berman, unpublished data).

† Observation validated by CGH in our lab.

‡ Observation validated by the PCR assay.

**Fig. 2:**
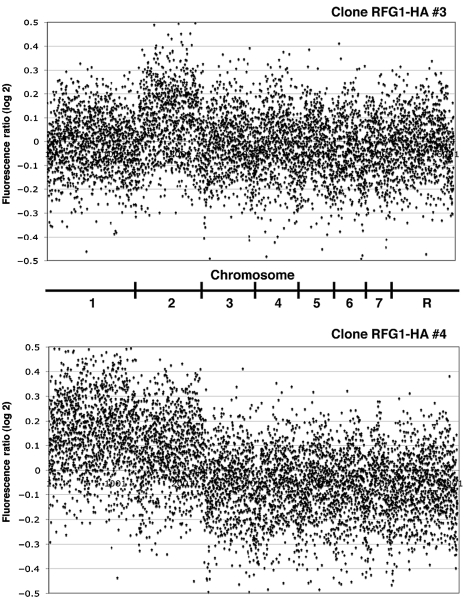
Example of different aneuploidies from two distinct colonies. These graphs represent the fluorescence ratios (log 2) from individual probes in a CGH comparing one of two colonies expressing the HA-Rfg1p transcription factor with a CAI4-pCaEXP empty-vector control strain that had previously been confirmed to have two copies of each chromosome. While the fold change in CGH should be expected to be at least 1.5-fold for a triploid vs. diploid comparison, we note that the quantarray software used to quantify our microarrays tends to underestimate fluorescence ratios.

**Fig. 3:**
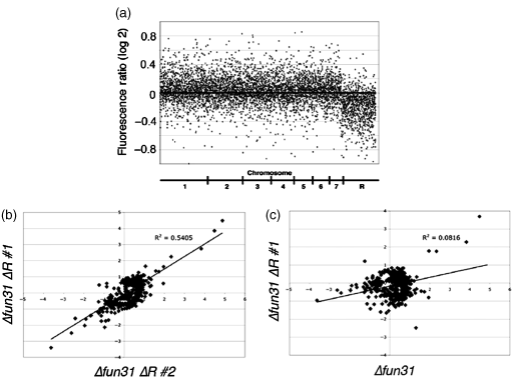
Aneuploidies can affect transcriptional profiles outside of the afflicted chromosomes. (a) Fluorescence intensities in a transcriptional profiling experiment of one of three Δ*fun31* mutants compared with a DAY185 control strain. Downregulation of Chr R genes is apparent. (b and c) Scatter plots showing the similarity of transcriptional profiles between the genes outside of Chr R in a comparison of two individual Δ*fun31* mutants lacking a copy of Chr R (b), or a Δ*fun31* mutant lacking a Chr R, when compared with a control strain without the Δ*fun31* mutation and an equal number of chromosomes. Spots represent the fluorescence ratios of 486 genes in Chr 1–7 that had a 1.5-fold change or more in at least one experiment. *R*^2^ values represent the similarities in the profiles between the two strains and indicate that the aneuploid strains produce profiles that are more similar to each other.

This analysis was conducted with transcriptional profiling data and not from CGH experiments, where genomic DNA from two strains is differently labeled and hybridized to microarrays. CGH on some strains has confirmed that the changes in transcript profiles are indeed the result of changes in gene copy number although it is difficult to determine precisely whether we are dealing with chromosome duplication, chromosome loss or some other type of karyotype rearrangement. In some cases, we noticed that individual transformed colonies can carry different aneuploidies. For example, Fig. 2 shows CGH data from two individual colonies of a strain, produced at the NRC-BRI, that express an HA-tagged version of the Rfg1p transcription factor. While both clones had the extra copy of Chr 2 normally found in the parental CAI4 strain, one of the clones also had an additional copy of Chr 1. Whether the extra Chr 1 arose from an independent duplication event or from a mixed population of Chr 2 and Chr 1/2 triploids in our CAI4 stocks has not been determined. Nevertheless, it should be noted that we failed to detect any Chr 1 bias in CGH experiments comparing our CAI4 and SC5314 stocks. Another case of colony-specific aneuploidy occurred during the production of Δ*fun31* mutants at Columbia University. As illustrated in Fig. 3a, transcriptional profiling on three independently isolated strains revealed that two of them seem to be missing a copy of Chr R (or have an extra copy of Chr 1–7). This experiment is also used to illustrate that a chromosomal aneuploidy can also affect gene expression patterns in the nonamplified chromosomes. A mutant vs. wild-type comparison of the transcriptional profiles of genes located on the nonaneuploid Chr 1–7 showed that the two strains with the Chr R aneuploidies were more similar to each other than to the strain with the correct number of chromosome copies (Fig. 3b and c). These observations, along with those produced by Selmecki *et al.* (2006), thus confirm that changes in the copy number of certain genes can result in compensatory changes in the expression profiles of other genes located outside of the afflicted chromosomes.

**Fig. 4:**
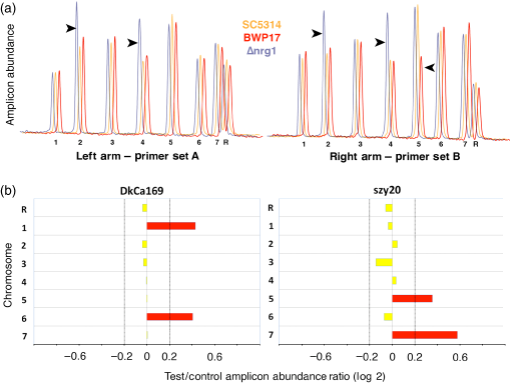
Aneuploidy detection with a multiplex PCR assay. (a) Bioanalyzer profiles of multiplex PCR reactions using primer set A (left panel) or primer set B (right panel). We used as templates genomic DNA from either a validated SC5314 control strain, a Δ*nrg1* strain (with extra copies of Chr 2 and 4) or the BWP17 strain carrying a heterozygous deletion on the right arm of Chr 5. Images of the profiles were scaled to similar sizes, thus allowing the identification of amplicons with a different abundance (arrowheads). In (b), the multiplex PCR assay was conducted with primer set A on genomic DNA from strains SC5314, DkCa169 (Legrand *et al.*, 2008) and SZY20 (Znaidi *et al.*, 2007). Graphs represent the mutant/SC5314 ratio of the median normalized peak heights on the *X*-axis and each chromosome on the *Y*-axis. A log_2_ ratio above 0.2 (in red) was considered to be significant and indicative of aneuploidy for these chromosomes.

In light of these results, we developed a multiplex PCR assay that can rapidly detect cases of chromosomal aneuploidy, the formation of isochromosomes or the loss of chromosome ends. Each assay consists of eight pairs of primers that are specific for unique regions near the left or the right arm of each chromosome (see Table 1). As illustrated in Fig. 4, the resulting PCR reactions produce eight amplicons of different sizes, one from each chromosome arm. The production of quantitative data requires some optimization specific for each lab and PCR machine, usually by varying the amount of template genomic DNA or the number of amplification cycles. For quantification purposes, we use an Agilent Bioanalyzer, a very precise capillary electrophoresis instrument that produces reproducible results in a very short amount of time. Identification of aneuploidies can be performed either by directly comparing the elution profiles (Fig. 4a) or by measuring normalized peak heights (Fig. 4b). In the examples shown in Fig. 4a, we can easily detect the extra Chr 2 and Chr 4 in the Δ*nrg1* strain as well as the small deletion in the right arm of the BWP17 Chr 5. In Fig. 4b, we can discern the extra Chr 1 and Chr 6 in strain DkCa169 that were observed by Legrand *et al.* (2008) as well as the additional Chr 5 and Chr 7 described by Znaidi *et al.* (2007) in the *TAC1/tac1*Δ::FRT SZY20 strain. We choose these strains because they represent examples of aneuploidies in seven out of the eight chromosomes, while BWP17 is an example of a deletion that only affects one arm.

## Discussion

We present evidence that the routine genetic manipulation of *C. albicans* often results in the acquisition of unwanted chromosomal aneuploidies. We have observed chromosome duplications in any one of eight chromosomes of *C. albicans*, with recombinant derivatives originating from either the CAI-4, RM1000, BWP17 or SN95 strains, and with strains produced by a variety of techniques including long-term treatment with Fluconazole, Ura-blaster-mediated gene deletion, the insertion of disruption cassettes produced by PCR, MPA^R^-flipping or the insertion of genes encoding tagged transcription factors at the *Rps1* site. Almost all the cases of chromosomal bias affected a different chromosome(s) than the actual site of the recombinant modification, which is to be expected because same-chromosome aberrations would have been easily detected as part of the regular Southern blotting controls that often follow strain production. Ever since we became aware of this problem, aneuploidy testing has been routinely applied in our lab and we have developed a rapid multiplex PCR assay that can cheaply identify chromosomal aberrations in less than a day. Although not every laboratory is expected to have access to a Bioanalyzer or similar equipment, band quantification from a regular gel is possible if the experimenter is skillful enough to detect a 50% increase in band intensity. Alternatively, the primer sequences included therein could easily be adapted into a quantitative PCR (qPCR) assay. In our hands, the PCR assay works fairly well in the detection of simple aneuploidies that affect one or even two chromosomes. Results with multichromosomal aneuploidies are much more difficult to interpret precisely; we can tell that something is wrong but matching peak heights to the afflicted chromosome is not always possible because we can not normalize the data. Finally, it should be noted that none of our assays can currently detect loss of heterozygocity (LOH). It would not surprise us if rates of unwanted LOH turned out to be as abundant as aneuploidies.

The phenotypic consequences of these chromosomal aberrations are difficult to assess without a direct comparison between an aneuploid and a nonaneuploid strain. The aneuploidy-dependent bias in transcriptional profiling data is generally very subtle, most notably because *C. albicans* is a diploid and an extra allele would thus increase gene dosage by 50%. Assuming an equivalent change in gene expression, this would only result in a 1.5-fold change in fluorescence ratio, which is the detection limit of most transcriptional profiling experiments. Consequently, we believe that most of the lists of significantly modulated genes are still valid. More worrisome and difficult to detect would be variations in gene expression patterns that would result from a change in transcription factor dosage, a phenomenon that was observed by Selmecki *et al.* (2006) with the Tac1p transcription factor.

In conclusions, based on our global microarray data analysis and general observations by the Montreal *Candida* research community, we believe that we are dealing with a fairly common phenomenon with a significant impact on *Candida* research. In light of these observations, we propose the following recommendations:

- The affected transcriptional profiles listed in Table 2 should not be used in global data analysis. The data currently in public databases, such as CGD and GEO, should be tagged appropriately.
- Important *Candida albicans* mutants and strains should be tested for aneuploidy, either by CHEF, CGH on DNA microarrays or by qPCR, before being used in subsequent experiments. For example, our lab recently produced 31 strains that express TAP-tagged transcription factors. These control experiments allowed us to eliminate six additional cases of aneuploidies (19%).
- During the production of *C. albicans* strains, multiple colonies should be isolated after every transformation to facilitate the isolation of a strain with the standard background karyotype.
- Finally, researchers should pay close attention to the culture stocks used for the construction of their recombinant strains. Ahmad *et al.* (2008) have identified CAF4-2 as a stable Ura^−^ derivative while we have tested the SN76, SN95, SN152 and SN148 strains (Noble & Johnson, 2005) and have found them to be initially free of aneuploidies.

## Statement

Re-use of this article is permitted in accordance with the Terms and Conditions set out at: http://www3.interscience.wiley.com/authorresources/onlineopen.html
